# Understanding the Role of Psychological Capital in Humorous Leadership-Employee Creativity Relations

**DOI:** 10.3389/fpsyg.2019.01636

**Published:** 2019-07-17

**Authors:** Zhengwei Li, Lihua Dai, Tachia Chin, Muhammad Rafiq

**Affiliations:** ^1^School of Management, Zhejiang University of Technology, Hangzhou, China; ^2^School of Economics and Management, Hangzhou Normal University, Hangzhou, China

**Keywords:** humorous leader, psychological capital, supervisor-subordinate dyadic tenure, work autonomy, employee creativity

## Abstract

This paper aims to examine how humorous leadership enhances employee workplace creativity from a novel angle of employee psychological capital (EPC). This study also explores the moderating roles of supervisor-subordinate dyadic tenure and work autonomy in the proposed model. Data from a sample of 355 supervisor-subordinate dyads working in an information technology enterprise in the People’s Republic of China was used to test the assumed moderated mediation model. The results indicate the positive relationship between humorous leadership and employee workplace creativity, which is partially mediated by EPC. Moreover, work autonomy significantly moderates the relationship between EPC and employee creativity. Humorous leadership has a significant effect on the extra role behavior of subordinates, leading to workplace creativity. The deliberate establishment of a humorous image by leaders may encourage subordinates to achieve creative goals. Combined with traditional management practices that emphasize the supportive behaviors of leaders, leaders can use humor to provide an open and friendly atmosphere for employees, thereby encouraging creativity in the workplace. Organizations should also place greater emphasis on employee work autonomy, giving employees enough flexibility on when and how they deal with their work; this could enhance the positive impact of other factors on employee workplace creativity. These findings carry implications for research on humorous leadership, EPC, and creativity.

## Introduction

A large body of literature has highlighted the significant role employee creativity plays in enhancing organizational innovation, effectiveness, survival, and competitiveness (Gong et al., 2009; Ghosh, 2015; Iqbal et al., 2015; Zubair and Kamal, 2015). Many scholars have explored how to drive employee creativity better. According to these studies, leadership styles can motivate employees (Iqbal et al., 2015; Goswami et al., 2016; Musinguzi et al., 2018) and enhance employee psychological capital (EPC), that is, the positive psychological state of their employees (i.e., hope, resilience, optimism, and efficacy) (Luthans et al., 2007), seems to be critically important in harnessing an employee’s creative potential. Moreover, the mediating role of EPC in the relationship between leadership style and employee creativity has been widely discussed. For instance, Rego et al. (2012) found that EPC mediates the association between authentic leadership and employee creativity. Gupta and Singh (2014) and Gong et al. (2009) highlighted the mediating impact of EPC on the relationship between transformational leadership and employee creativity. Zhang and Bartol (2010) demonstrated the intervening effect of employee psychological state on the relationship between empowering leadership and employee creativity. Noticeably missing from the literature is an examination of “humorous leadership” and how it relates to EPC.

A leader’s sense of humor might facilitate followers to think “outside of the box” and achieve greater creativity (Cooper, 2008). We argue that a leader’s sense of humor may be of particular significance in motivating employee creativity in China. Because Chinese culture values high power, respecting for those in power is a prominent cultural characteristic among Chinese people (Nonaka and Zhu, 2012; Chin and Liu, 2015). As such, employees consider Chinese leaders to be unchallengeable authorities; leaders are perceived to take their work seriously and maintain a certain distance from their subordinates (Chan, 2014; Chin and Liu, 2015; Du et al., 2019). Scholars claim that humorous leadership may activate subordinates’ creativity (Lang and Lee, 2010; Wood et al., 2011; Pundt, 2015; Yam et al., 2018), as humor allows for the playful combination of ideas that appear incongruent at first glance; through this, innovation emerges. Nevertheless, limited research has addressed the importance of humorous leadership in China, despite the wide recognition of its influence on employee innovation performance in western countries (e.g., Avolio et al., 1999; Vecchio et al., 2009; Robert and Wilbanks, 2012). To fill the gaps mentioned above, the current study aims to explore the relationships among humorous leadership, EPC, and employee creativity.

Although humorous leadership appears to be a popular kind of leadership, is it always helpful? We argue that humor is not a panacea. That is to say, although a leader’s sense of humor may help to increase positive effect among subordinates, it may also increase the deviance of the subordinates. Because it is easier to communicate with a humorous leader, employees are more likely to break the company rules, and this will inevitably lead to confusion. Furthermore, in her qualitative study, Holmes (2007) reported that humorous comments during team meetings kept the idea generation process going, although humorously presented ideas were often unrelated to the actual solution. Evidence indicates that humorous leader often appears to be ineffective and unprincipled; this leaves a bad impression on employees who see their leader as a role model. The impact of humorous leadership on employee creativity is not created out of thin air; it is affected by some contingency factors. Therefore, the main purpose of this study was to build and test a theory that addresses the connection between humorous leadership and workplace creativity, including several important intervening variables.

In building a model linking humorous leadership and employee creativity, we further drew on Resource Conservation Theory (Hobfoll, 1989) and Emotional Event Theory (Weiss and Cropanzano, 1996) to posit a mediating mechanism that can potentially explain the link between humorous leadership and employee creativity; specifically, EPC. EPC, defined as “an individual’s positive psychological state of development,” is characterized by: “(1) having confidence and self-efficacy to take on and put in the necessary effort to succeed at challenging tasks; (2) making a positive attribution about succeeding now and in the future; (3) persevering toward goals and, when necessary, redirecting paths to goals in order to succeed; and (4) when beset by problems and adversity, sustaining and bouncing back and even beyond to attain success” (Luthans et al., 2007). Thus, we explored the extent to which humorous leadership works through EPC to ultimately influence employee creativity. Although “creativity” can be used to describe both an outcome and a process, in this study, we use the word in the outcome sense–that is, to denote the extent to which novel and useful ideas are produced.

Finally, we proposed and tested two potentially important moderators of the relationship between humorous leadership and employee creativity: work autonomy and supervisor-subordinate dyadic tenure. Work autonomy refers to the extent to which employees can make decisions about their own work, such as when they work, who they work with, and so on. Supervisor-subordinate dyadic tenure refers to the duration of time an employee has worked together with his direct leader.

Overall, our purpose was to build a theory by conceptually and empirically linking Resource Conservation Theory and Emotional Event Theory to provide a more comprehensive understanding of the humorous leadership phenomenon as it relates to employee creativity.

## Theory and Hypotheses

**Figure 1:**
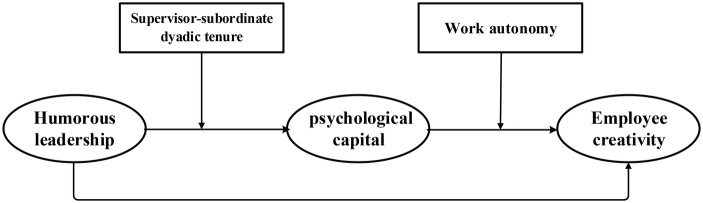
Hypothesized model.

In this section, we trace the development of our overall research model by first exploring the general nature of humor and humorous leadership as it relates to creativity. Next, we investigate how humorous leadership influences EPC, as delineated by Luthans et al. (2007), including consideration of a potential moderator: supervisor-subordinate dyadic tenure. We then examine the influence of EPC on employee creativity. As part of these arguments, we incorporate work autonomy as a moderating variable to help explain how leaders can affect the extent to which EPC influences employee creativity. The hypothesized model is depicted in Figure 1.

### The Nature of Humor

The word “humor” comes from the Latin word “homorem,” with the original meaning of “liquid” or “fluid.” Broadly defined, a sense of humor refers to an individual trait-like tendency to use or display behaviors, attitudes, and abilities relating to amusement during social interactions (Martin, 2001; Al Obthani et al., 2013). Research on humor has gradually entered into the field of organizational behavior. Sense of humor is often said to be a critical component of successful leadership (Sobral and Islam, 2015). Sense of humor in an organization may also be a means of relaying verbally expressed ingratiation behavior. Specifically, a humorous sender shares an event with others for the purpose of entertainment and pleasant, the recipient can feel this is intentional (Cooper, 2005).

The evolution of the concept of humor has mainly involved different views as to whether humor is a behavior or trait. The trait view holds that personal humor is a stable personality trait related to personal experiences and attitudes toward life. This view emphasizes that an individual’s sense of humor is stable and consistent; it does not change greatly due to certain events or in special periods. It is mainly manifested in the behavioral tendency of individuals to actively capture, discover, and create humor in the workplace. However, the behavioral view argues that humor in the workplace is deliberately initiated to purposefully deliver interesting events to the recipient. The behavioral view emphasizes that the initiator intentionally creates and then sends a signal with humor, and then through the dissemination of the organizational media, the receiver analyzes and decodes. Finally, the receiver responds to the humor, such as with laughter, happiness, and other information dissemination processes.

At present, the behavioral view of humor is much more popular among scholars. Humor is regarded as a form and process of interpersonal communication in an organization. Humorous leadership refers to a leader, who is always consciously entertaining his subordinates by sharing interesting things (Robert et al., 2016). According to Martin et al. (2003), there exist four different styles of interpersonal humor: affiliative, self-enhancing, aggressive, and self-defeating humor. Since the 1980s, researchers have explored the impact of workplace humor on work efficiency. With consideration for the various dimensions of humor, many scholars have studied the relationship between humorous leadership and employee performance, mental health, work satisfaction, and so on (e.g., Avolio et al., 1999; Vecchio et al., 2009; Kim et al., 2016).

Research on leadership style is a hot topic in the study of organizational behavior. As an important type of leadership style, humorous leadership has attracted the attention of an increasing number of scholars. Empirical research has demonstrated that humor is closely related to organizational innovation, socialization, employee relationships, job satisfaction, work engagement, team cohesion, and so on (Bizi et al., 1988; Wood et al., 2011; Yam et al., 2018). Successful humor in an organizational setting can add the quantity and quality of effective communication within the team, alleviate boredom and frustration at work as well as improve the relationship between leader and employees, thus improving overall work efficiency.

Adriaenssens et al. (2015) investigated the relationships between various leadership styles and employee performance. The results showed that positive leadership styles, such as transformational leadership and contingent leadership, were positively related to humor. On the contrary, laissez-faire leadership was negatively related with humor. For positive leaders, the use of humor can make them look more confident and glamorous, allowing them to send positive signals to employees, so as to enhance team cohesion and create good organizational culture; thus, improving organizational innovation and team performance. For the sake of distinction, humor in this article only considers the positive aspects, including affiliative humor and self-enhancing humor; this paper does not take two negative kinds of humor (aggressive and self-defeating humor) into consideration.

### Humorous Leadership and Employee Creativity

In general, creativity in the workplace is defined as the production of novel and useful ideas or solutions (Amabile, 1988; Shalley, 1991; Oldham and Cummings, 1996). According to this definition, both novelty and usefulness are necessary elements for an idea or product to be judged as creative (Zhou and George, 2003). In the context of creativity at work, an idea or product which only displays novelty or usefulness is not enough; a novel idea that has no potential value cannot be regarded as creative, nor can a useful product that is not significantly different from already available ones (Chang et al., 2015).

A survey of 329 Fortune 500 CEOs performed by Fortune magazine found that 97% of CEOs agree with the importance of humor in business; these findings call for CEOs to cultivate a greater sense of humor (Chen and Chen, 2011). So, how do humorous leaders influence employee innovation behavior? According to Benign Violation Theory (BVT; McGraw and Warren, 2010), it is necessary and beneficial to break some benign rules. Briefly, BVT suggests that the display of humor often necessitates a benign norm violation. Leaders’ humorous behavior conveys to other members of the organization that breaking some existing rules is acceptable; thus, subordinates are more likely to increase workplace violations (Robinson and Bennett, 1995). In their eyes, violations are not “unforgivable,” but safe to try. This kind of workplace violation allows employees continually explore new ideas without sacrificing the original production model; thus, increasing the possibility of innovation.

Evidence indicates that the atmosphere in the workplace plays an important intermediary role between human resource practice and organizational performance (Delaney and Huselid, 1996; Tsai et al., 2015). If employees feel their company attaches great importance to innovation, this will give them the belief that conducting innovative actions is encouraged; even if their innovation fails, they will not be punished, the subjective efforts of the employees will be stimulated and they will try innovative methods actively (Hommel et al., 2011). Conversely, if employees believe that the organization does not value innovation as they say, or that they will suffer big losses if they fail, then employees will be unlikely to try innovative and new processes or methods (Wang and Wang, 2018). Humorous leadership plays an important role in the creation of an organizational innovation atmosphere; in such an atmosphere, subordinates feel free to develop, communicate, and implement their ideas without any fear of negative consequences (Carmeli et al., 2010). Humorous leaders may make funny comments about upcoming mistakes, and subordinates face idea implementation with a more lighthearted and playful approach.

Further, humor is one kind of creativity itself; that is, humorous leadership gives way to creativity and idea generation as part of innovative behavior. According to BVT, incongruity is a cognitive element of humor (Yam et al., 2018). This means the first condition of humor is that at least two situational features are cognitively incompatible. Situations that violate expectations, but are simultaneously perceived as being normal overall, are typically perceived as humorous (Veatch, 1998). Thus, humorous leaders are models of person with creativity. Emotional Event Theory argues that the mental state of a leader can be perceived by the employees as an environmental impact, which in turn affects the attitudes and behaviors of the employees (Scott and Bruce, 1994; Weiss and Cropanzano, 1996). Humorous leader will positively influence subordinates, who see him as a role model, and thus, subordinates will introduce new process and ideas so as to find a common language with their leader (Lebedeva et al., 2018). Hence, humorous leadership has a positive effect on employee creativity:

*H1:* Humorous leadership is positively related to employee workplace creativity.

### Humorous Leadership, Employee Psychological Capital, and Employee Creativity

Studies have shown that individual cognitive factors have a significant impact on employee innovation behavior (Tierney and Farmer, 2011). The influence of leadership on employee behavior is often not direct, but is generated by the internal psychology and cognition of employees (Shin and Zhou, 2003; Cho and Dansereau, 2010; Walumbwa et al., 2010). EPC, defined as “an individual’s positive psychological state of development” includes four aspects: confidence (self-efficacy), optimism, hope, and resilience (Luthans et al., 2007). Although the study of EPC has mostly focused on its structural dimensions and its connotation level, we still have reason to believe that there is a close relationship between humorous leadership and employee EPC (Gupta and Singh, 2014; Wang et al., 2018).

The innovation process is full of complexity and uncertainty; employees need to have strong confidence and self-efficacy (Tierney et al., 1999; Silla and Gamero, 2018; Rafiq et al., 2019). Humorous leader garners trust and confidence from their subordinates through funny words and deeds. By providing cognitive, emotional, and ethical assistance to their employees, humorous leader enables them to develop their own abilities and promote their self-confidence. Through observing exemplary behaviors in a respectful manner, employees may develop greater confidence in their abilities to pursue goals (Rego et al., 2012). Individuals with higher self-efficacy are more likely to take risks and engage in challenging tasks, and are therefore more likely to use creative methods to solve problems (Gong et al., 2009). Participative goal setting enhances an individual’s willingness and ability to design hope pathways. Breaking down difficult goals into smaller and more manageable milestones can also enhance hope in employees (Gupta et al., 2011). Humorous leaders usually have a good relationship with their subordinates, and tend to set work goals together with their subordinates. Further, humorous leaders increase their subordinates’ level of optimism by creating a supportive work environment.

By providing positive feedback to their subordinates and expressing confidence in their abilities, humorous leader can motivate their employees to look on the bright side of things, redirect his employees away from the negatives and focus on the positives and available opportunities (Gupta et al., 2011). Through some fixed-themed training, humorous leader can demonstrate and teach realistic optimism to employees, and through this process, innovative behaviors among subordinates are promoted. Positive feedback and encouragement in the work from the leader can help enhance employees’ resilience (Luthans et al., 2007). Resilience can also be enhanced by altering the perceived level of risk and generally fostering self-enhancement and development (Avey et al., 2009). In the face of pressure or adversity, humorous leader can help subordinates to respond positively. Therefore, when the subordinates face difficulties, they will not only persist, but ultimately succeed; this will help to improve the level of resilience in subordinates (Avolio and Walumbwa, 2006).

The innovation process is so difficult and uncertain that it requires employees to have an unwavering internal drive to transcend current challenges and setbacks in order to adapt to the changing environment. Resilient employees can be unyielding in a dynamic environment and can meet the needs of creative problem solving. Self-efficient employees are more confident in their innovative ideas and are more willing to propose novel ideas in the workplace. While self-efficiency means that employees have the power to do innovative things, high levels of hope indicate that employees can do challenging work in different ways (Luthans et al., 2007). In order to obtain leadership support for their innovative ideas and behaviors, employees with high level of hope develop practical solutions based on the leader’s hobby, and exhibit determination to overcome risks and challenges brought about by innovation failures. Optimistic individuals can are more likely to control their own destiny and face difficulties as well as failures more calmly. They often have positive expectations of themselves, and thus, are more easily to achieve innovative behavior (Sweetman et al., 2011). Based on the above arguments, we hypothesize that:

*H2:* Humorous leadership behavior is positively related to EPC.

*H3:* EPC is positively related to employee creativity.

*H4:* EPC mediates the relationship between humorous leadership and employee workplace creativity.

### The Moderating Role of Supervisor-Subordinate Dyadic Tenure

While overall, we expect humorous leadership to positively influence employee EPC, there is some evidence that employees differ in the extent of EPC, even in the same context of humorous leadership. To assess this prospect, we draw on Emotional Event Theory (Weiss and Cropanzano, 1996), according to which, the mental state of the leader can be perceived by employees as an environmental impact, which in turn affects the attitudes and behaviors of employees. As managers of the workplace, leaders are responsible for leading employees to complete required tasks. Their mental state and leadership behavior is an important working background against which the employees carry out their daily work; thus, the mental state and leadership of a leader have important influence on the work attitudes and behaviors of employees (Podsakoff et al., 1990; Niehoff and Moorman, 1993).

As mentioned earlier, the supervisor-subordinate dyadic tenure is defined as the duration of time that an employee has worked together with his direct leader. Individuals can achieve a sense of belonging and self-realization through communication and interaction, thus increasing EPC (Quinn and Dutton, 2005). According to Emotional Event Theory, the behavior and performance of employees are the results of the comprehensive influence of the surrounding environment; the behavior and mental state of the leader is a very important factor in the surrounding environment of the employees. The longer of the supervisor-subordinate dyadic tenure, the greater positive interactions and emotional exchanges between the leader and employees are. Further, with greater supervisor-subordinate dyadic tenure, the leader will have more trust and support in their employees (Gkorezis et al., 2011); in turn, the employees will appreciate this and engage in more positive organizational behaviors, such as working harder, taking on more work commitments, and engaging in innovative work methods. All of the organizational behavior mentioned above can contribute to a gaining EPC of employees.

Robert et al. (2016) found that only when an employee works with a leader for a long duration of time will they establish a relationship with high quality exchanges. The longer an employee works with his direct leader, the more they can get to know each other, including each other’s behavioral habits, work style, and even hobbies. This will eliminate and avoid misunderstandings caused by insufficient or inappropriate communication. Further, frequent interactions between the leader and employees can help the employees gain psychological power from the guidance of their leader. EPC, which can be increased after training and intervention, is one such important kind of positive psychological power. In short, supervisor-subordinate dyadic tenure provides a valuable time basis and support for positive communication between leaders and employees; that is, the supervisor-subordinate dyadic tenure is longer, the positive relationship between humorous leadership and EPC is stronger. On the contrary, the supervisor-subordinate dyadic tenure is shorter, the positive association between humorous leadership and EPC is weaker. Accordingly, we propose that:

*H5:* Supervisor-subordinate dyadic tenure moderates the relationship between humorous leadership and EPC.

Based on the discussion above, combined with H4 and H5, we further infer that the mediating role of EPC on the relationship between leader’s sense of humor and employee creativity can be strengthened in employees who have a longer supervisor-subordinate dyadic tenure. In other words, the mediating role of EPC is moderated by supervisor-subordinate dyadic tenure; thus, we propose a moderated mediation model. Our specific proposition is as follows: supervisor-subordinate dyadic tenure positively moderates the mediating role of EPC on the relationship between humorous leadership and employee creativity. Specifically, when employees and leaders work together for a longer duration of time, the mediating role of EPC is relatively strong; however, when employees and leaders work together for only a short duration of time, the mediating role is relatively weak.

### The Moderating Role of Work Autonomy

Although there are conceptual and empirical reasons to expect that an employee with high EPC will be more creative in the workplace, EPC, by definition, leaves an employee with considerable scope. As mentioned above, creative behavior in the workplace is not clearly listed as a job responsibility, and employees may even bear some risks and losses while being creative; thus, employees face external pressure when they engage in creative behavior. According to Resource Conservation Theory (Hobfoll, 1989), when individuals feel pressure, they will actively seek resources to alleviate the psychological disorder caused by stress; ways in which they may seek such resources include obtaining control and autonomy over their work. Work autonomy, as a key indicator of job characteristics, refers to the degree to which employees can independently control and decide on their working methods, work arrangements, and work standards (Breaugh, 1989). Llopis and Foss (2016) argued that employees with more job autonomy have greater freedom to decide which tasks to perform, how the work will be done and how work contingencies are to be handled.

Employees with a high degree of autonomy in their work can decide their own work style and schedule; this can intrinsically motivate employees and meet their need for a sense of belonging. When work autonomy is improved, employees have clearer work responsibilities and obligations. All of these factors can lead to workplace creativity. Further, work autonomy can improve the internal perceptions of employees by improving their mental state and work performance (Hackman and Oldham, 1976); work autonomy may also play a role in regulating the relationship between individual emotions and behaviors (Bizzi and Soda, 2011; Wang et al., 2018). Employees with a high degree of work autonomy can participate in the decision-making process; they also have access to job-related information to the fullest extent, and are therefore less affected by contextual factors. This can accelerate the process of transformation from EPC to workplace creativity. Accordingly, we propose that:

*H6:* Work autonomy moderates the relationship between EPC and workplace creativity.

Based on the discussion above, combined with hypothesis H4 and H6, we further infer that the mediating role of psychological capital between humorous leadership and employee creativity is influenced by work autonomy. In other words, the mediating role of psychological capital is moderated by work autonomy. Therefore, we propose the following assumption:

Work autonomy positively moderates the mediating role of psychological capital between humorous leadership and employee creativity. Specifically, when employees have more autonomy in workplace, the mediating role of psychological capital is relatively strong; otherwise, the mediating role is relatively weak.

## Materials and Methods

### Research Setting and Participants

This study was conducted in a major information technology (IT) company headquartered in the People’s Republic of China (PRC). Participants were professional-level employees whose work required substantial creativity in order to be effective. The direct supervisor of each participating employee was also recruited for this study. We used a pairing survey to collect data from the two sources in order to reduce common method biases. The entire survey was translated from English into Chinese and then back-translated into English by two independent bilingual individuals to ensure equivalency of meaning. Separate questionnaires were designed for direct leaders and employees. The employee questionnaire (questionnaire A) collected data about the employee’s evaluation of his direct leader’s sense of humor, as well as EPC, work autonomy, and personal information related to the employee. The direct leader questionnaire (questionnaire B) contained an evaluation of employee creativity and collected information related to the team and the leaders themselves.

Before commencing the survey, we contacted the company’s human resources (HR) department and got a list of 150 teams of this company. Then, we coded all the questionnaires and matched each leader questionnaire (questionnaire B) with the employee questionnaire (questionnaire A). With the name list of the employees in the 150 teams above, we used a simple random sampling method to choose 600 employees to participate in our survey. We distributed a total of 600 employee questionnaires (questionnaire A), and received 423 valid ones, yielding a response rate of 70.5%. Then, we distributed questionnaires (questionnaire B) to the corresponding supervisors of the 423 employees who responded and obtained 355 valid supervisor responses at last. Through sorting out of these questionnaires, we found the 355 employees in our survey were from 83 teams. So, we got 355 pairs of valid samples in our survey in total.

At last, we obtained a total of 355 pairs of leader-member questionnaires, including 83 leaders and 355 employees. Each of these 355 employees was evaluated by his direct leader. Similarly, all the leaders were evaluated by their employees separately. The majority of the participating employees were between 20 and 25 years of age. The employees in this age group accounted for 75.2% of the total number of participants. The average age of the leaders was 27–29 years of age. Among the employees, there were 197 males, accounting for 55% of the total number of employees. Male leaders accounted for the majority of the leaders; 83 leaders participated in the survey, 51 of which were males, accounting for 61.4% of the total number of leaders.

### Measures

#### Leader’s Sense of Humor

We measured leader sense of humor with a 7-item scale developed by Thorson and Powell (1993). The participants responded using a 5-point scale ranging from 1 = “strongly disagree” to 5 = “strongly agree.” Sample item: “My leader uses humor to entertain coworkers.” Cronbach’s *α* was 0.92.

#### Employees’ Psychological Capital

EPC was measured with a 24-item scale developed and validated by Luthans et al. (2007). The scale has four sub-scales, namely, hope, resiliency, optimism, and self-efficacy; each scale is measured with six items. Items were measured on a 5-point Likert scale (1 = “strongly disagree” to 5 = “strongly agree”). Sample items include “I feel confident analyzing a long-term problem to find a solution” (self-efficacy); “I always look on the bright side of things regarding my job” (optimism); “If I should find myself in a jam at work, I could think of many ways to get out of it” (hope); and “I usually take stressful things at work in stride” (resiliency). Cronbach’s *α* was 0.97.

#### Work Autonomy

A 7-item scale adapted by Kirmeyer and Shirom (1986), with minor modification, was used to assess perceived work autonomy. The scale asked participants to indicate the extent to which they agreed with the statement about the freedom they feel regarding to their work, such as when they work, with whom they work, how they finish their work, and so on. Respondents rated their perceived extent of freedom on a 5-point Likert-type scale ranging from 1 (strongly disagree) to 5 (strongly agree). Sample item: “To what extent do you feel you have latitude to decide when to take breaks”. Cronbach’s *α* was 0.92.

#### Employee Creativity

Employee creativity was measured with a 13-item scale developed by Zhou and George (2003). Leaders responded on a 5-point scale ranging from ‘not at all characteristic’ to “very characteristic”. Sample item: “He (the employee) is a good source of creative ideas”. Cronbach’s *α* was 0.94.

#### Supervisor-Subordinate Dyadic Tenure

Supervisor-subordinate dyadic tenure was measured as the duration of time, the employee had worked with his direct leader.

#### Control Variables

We controlled for three demographic variables in our analyses as previous research has found these to be correlated with employee creativity (e.g., Zhou and George, 2003). Age was measured in years. Gender was measured as a dichotomous variable coded as 0 for male and 1 for female. Education was measured as the number of years of post-high-school education.

### Measures Validation of Measures

As reported above, the Cronbach’s alphas for all multi-item scales were greater than 0.92, indicating good reliability. Next, convergent and discriminant validity were evaluated to examine the measurement model. The factor loadings λ of all multi-item constructs were higher than 0.60, the average variance extracted (AVE) for each variable was greater than 0.50, and the composite reliabilities (CR) were greater than 0.8, indicating that each measurement construct had great convergence validity (Fornell and Larcker, 1981). In addition, as shown in Table 1, the square root of AVE of each variable on the diagonal was greater than the correlation coefficient of the variables presented in the same row or the same column. Therefore, the variable construction of the four multi-index measurements had good discriminant validity (Fornell and Larcker, 1981).

**Table 1:** Descriptive statistics, correlations, and reliabilities (*N* = 355).

		1	2	3	4	5	6	7	8
1	Age	NA							
2	Gender	0.18^**^	NA						
3	ED	0.19^**^	0.05	NA					
4	HL	0.01	0.15^**^	0.03	0.79				
5	SDT	0.29^**^	0.07	0.05	0.07	NA			
6	EPC	0.13^*^	0.15^**^	0.14^**^	0.33^***^	0.09	0.76		
7	WA	0.12^*^	0.10	0.11^*^	0.20^***^	0.07	0.57^***^	0.80	
8	EC	0.13^*^	0.10	0.10	0.29^***^	0.20^***^	0.36^***^	0.30^***^	0.75
	Mean	22.12	0.44	1.80	4.07	1.27	3.97	3.30	3.28
	SD	1.94	0.50	1.58	0.75	0.65	0.69	0.92	0.70

*The square roots of AVE for the four multi-index constructs are given in parentheses on the diagonal*.

* p < 0.05;

** p < 0.01;

*** *p < 0.001*.

*EC, employee creativity; ED, education; EPC, Employees’ psychological capital; HL, humorous leadership; NA, not available; SDT, supervisor-subordinate dyadic tenure; WA, work autonomy*.

### Analysis

To test our hypothesized moderated mediation model, we used the SPSS macro PROCESS (version 3.2) developed by Hayes et al. (2017). This allowed us to conduct bootstrapping examinations for moderation and moderated mediation in order to calculate the indirect impact of humorous leadership on employee creativity *via* EPC, at different levels of supervisor-subordinate dyadic tenure and work autonomy. Before using the SPSS macro PROCESS, all measures in the interaction terms were mean-centered (Aiken et al., 1991).

## Results

The means, standard deviations, and correlations for each study variable are shown in Table 1. All the correlation results were in the expected direction.

Results presented in Table 2 show that, after controlling for the impact of demographic variables, humorous leadership was positively related to employee creativity (*β* = 0.28, *p* < 0.001) and EPC (*β* = 0.32, *p* < 0.001), and EPC was also positively related to employee creativity (*β* = 0.33, *p* < 0.001). These findings are in line with H1, H2, and H3.

**Table 2:** The mediation role of EPC.

Variables	EPC		Employee creativity			
	Model 1	Model 2	Model 3	Model 4	Model 5	Model 6
Age	0.13^*^	0.14^**^	0.14^*^	0.14^**^	0.09	0.11^*^
Gender	0.13^*^	0.08	0.09	0.04	0.04	0.02
ED	0.14^**^	0.14^**^	0.12^*^	0.12^*^	0.08	0.09
HL	–	0.32^***^	–	0.28^***^	–	0.20^***^
EPC	–	–	–	–	0.33^***^	0.27^***^
Adjusted *R*^2^	0.04	0.14	0.03	0.11	0.13	0.16
*F*	6.40^***^	15.71^***^	4.64^**^	11.52^***^	14.48^***^	14.92^***^

* p < 0.05;

** p < 0.01;

*** *p < 0.001*.

*EC, employee creativity; ED, education; EPC, employees’ psychological capital; HL, humorous leadership*.

Table 2 shows the results of our H4 mediation hypothesis. It can be seen that when we included both humorous leadership and EPC as independent variables and employee creativity as the dependent variable, EPC had a positive impact on employee creativity (*β* = 0.27, *p* < 0.001) while humorous leadership continued to have a significant impact on employee creativity, although the predictive effect was significantly attenuated (*β* = 0.20, *p* < 0.001). This indicates that there is a partial mediation effect. Finally, the bias-corrected percentile bootstrap method indicated that the indirect impact of humorous leadership on employee creativity through EPC was significant, effect = 0.18, with a 95% CI of [0.0002, 0.0889]. Thus, H4 was supported.

**Figure 2:**
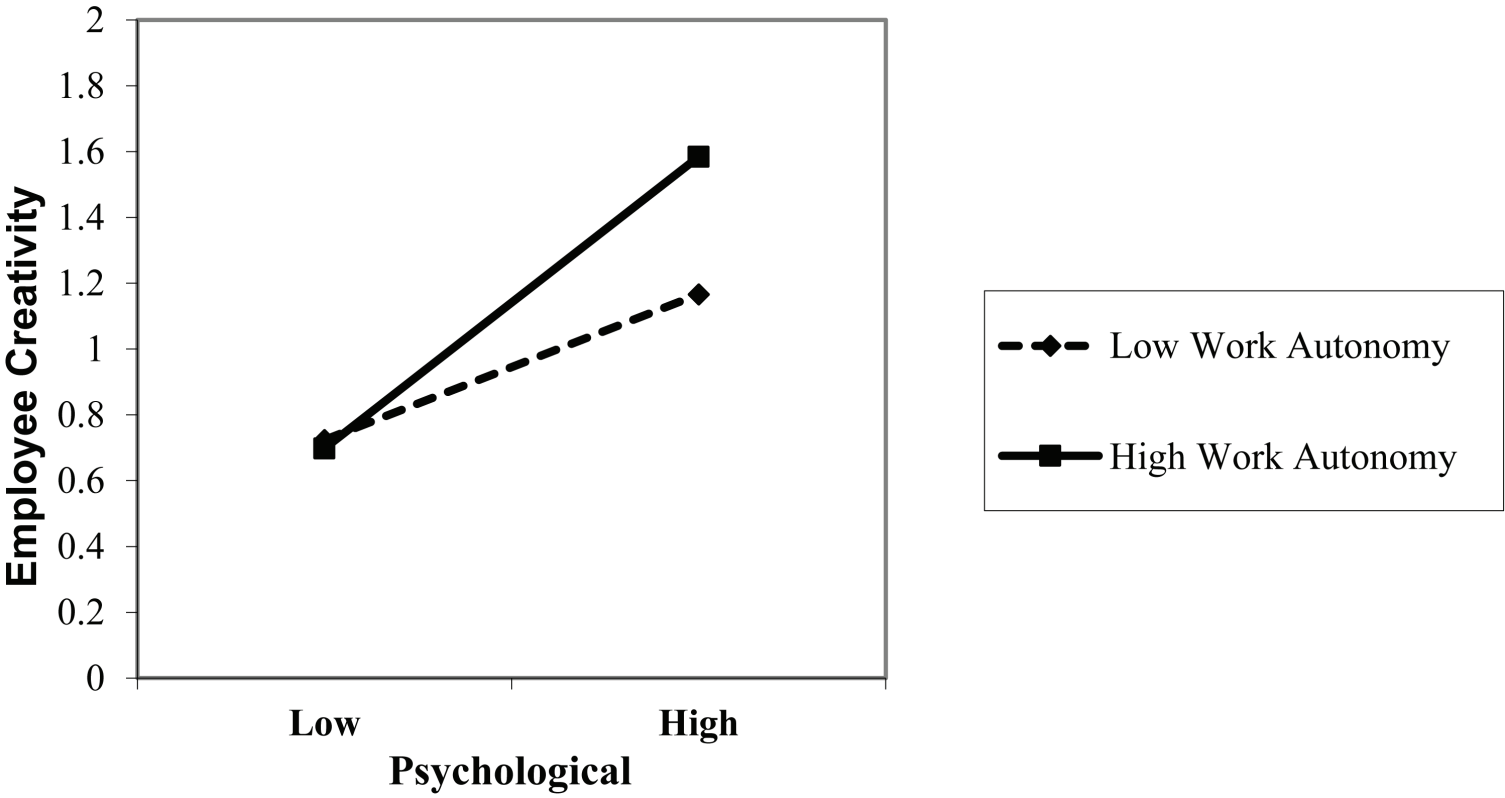
Moderation effect of work autonomy.

Table 3 shows the results of our H5 and H6 moderation hypotheses. As can be seen below, the effect of the interaction between humorous leadership and supervisor-subordinate dyadic tenure on EPC was not significant; thus, H5 was not supported. On the other hand, the interaction between EPC and work autonomy was found to positively predict employee creativity (*β* = 0.14, *p* < 0.05), even when controlling for demographic variables. Figure 2 shows that when the level of work autonomy is high, the employee’s creativity increases more, and when the level of autonomy is low, the increase is small. It can be intuitively seen that work autonomy strengthens the positive effect of psychological capital on employee creativity. Thus, overall, H6 was supported.

**Table 3:** Moderation effect of the model.

Variables	Moderation effect					
	Model 1	Model 2	Model 3	Model 4	Model 5	Model 6
Age	0.13^*^	0.11	0.08	0.08	0.04	0.034
Gender	0.13^*^	0.13^*^	0.13^*^	0.13^*^	0.09	0.085^**^
ED	0.14^**^	0.14^**^	0.14^**^	0.12^*^	0.07	0.063
HL		0.32^***^	0.33^***^			
SDT		0.04	0.02			
EPC					0.26^***^	0.33^***^
WA					0.14^*^	0.13^*^
HL^*^SDT			0.053			
PC^*^WA						0.14^*^
Adjusted *R*^2^	0.05	0.141	0.141	0.03	0.142	0.15
*F*	6.39^***^	12.65^***^	10.70^***^	4.64^***^	12.72^***^	11.74^***^

* p < 0.05;

** p < 0.01;

*** *p < 0.001*.

*ED, education; EPC, employees’ psychological capital; HL, humorous leadership; SDT, supervisor-subordinate dyadic tenure; WA, work autonomy*.

Finally, PROCESS macro in combination with the Bootstrap method proposed by Hayes et al. (2017) were used to further examine the conditional indirect effect of humorous leadership on employee creativity through EPC, at two levels of employee work autonomy (+1 SD above the mean and −1 SD below the mean). In Table 4, results showed that the conditional indirect effect of humorous leadership on employee creativity *via* EPC was 0.11 with a 95% CI of [0.050, 0.189] when the level of work autonomy was high, versus 0.05 with a 95% CI of [0.017, 0.101] when the level of work autonomy was low. Additionally, the moderated mediation index was also significant 0.03, with a 95% CI of [0.0058, 0.0601].

**Table 4:** Bootstrap test results of moderated mediating effect.

	Indirect effect					Moderated mediating effect			
Dependent variable	Work autonomy	Effect	SE	95% LLCI	95% ULCI	INDEX	SE	95% LLCI	95% ULCI
Employee Creativity	Low	0.05	0.02	0.017	0.101	0.03	0.014	0.0058	0.0601
	High	0.11	0.03	0.050	0.189				

*N = 355, confidence interval 95%*.

## Discussion

In this paper, we built and tested a conceptual model that uniquely combines humorous leadership theory with creativity theory. Although a number of studies have investigated relationship between leadership style and creativity (Zhou and George, 2003; Amabile et al., 2004), humorous leadership has been surprisingly absent from consideration. Yet, as we have argued and uniquely modeled, there are strong theoretical reasons to expect humorous leadership to be well positioned to influence fundamentals underlying employee creativity, a contention that we have empirically supported here. Our results support suggestions of creativity scholars that leadership approaches may be an effective means for encouraging employee creativity.

Our research makes a number of important theoretical contributions to the literature on humor and creativity. Using the theory of emotional events, this paper introduces EPC as a mediator variable, linking the behavior of the leaders with employee psychology and employee behavior. At the same time, we also introduce supervisor-subordinate dyadic tenure and employee work autonomy as two situational factors to study their moderating effects on the model. Based on the empirical findings, it is confirmed that humorous leadership has a significant positive impact on employee creativity, and EPC plays a partial mediation role. At the same time, employee work autonomy positively moderates the relationship between EPC and employee creativity. When employees have a high level of work autonomy, EPC has a greater impact on employee creativity. The findings suggest that this is a moderated mediation model, which indicates that employee work autonomy not only moderates the positive relationship between EPC and employee creativity, but also moderates the mediation effect of EPC on the relationship between humorous leadership and employee creativity. When employees have a high level of work autonomy, EPC has a stronger mediating effect on the link between humorous leadership and employee creativity.

Our theoretical model also has some practical implications for companies especially for managers. Firstly, in encouraging employee creativity, leadership does matter. Specifically, our results suggest that humorous leadership has the capacity to positively influence employee EPC, and it is an important role in influencing employee creativity. Thus, in order to improve employee creativity, one effective way is to enhance the psychological state of the employees, including level of self-efficacy, optimism, hope, and resilience. For managers, it is desirable and even necessary to become humorous in the workplace. Further, work autonomy acts as a situational factor, influencing the link between employee EPC and employee creativity. Managers need to make a conscious effort to effectively improve the work autonomy of employees. With high level of autonomy at work, employees can freely determine issues at work such as when to rest, who they would like to work with, etc.

Till now, few scholars have combined humorous leadership styles with the psychological capital of employees to explore employee creativity like us. It is easy to see that leadership style is an important factor for the work attitude and behavior of employees (especially creativity in the workplace), and the psychological state of employees is the direct influence of behavior. What is more, the theory of Emotional Events also proves this path from which employees accrue creativity with the help of humorous leader. In practice, the results of our research are also meaningful. By following the advice given in the article, we can expect companies effectively improve employees’ creativity in the workplace.

Like any study, this study is not without limitations. Firstly, self-report methods were used to collect data from employees, raising the possibility of same-source bias. Since the measured constructs (EPC, supervisor-subordinate dyadic tenure, and work autonomy) address the individual’s internal states, we would argue that it is logical to collect the data from the participants themselves. A mitigating factor is that the ratings of employee creativity were collected from each employee’s direct leader, and leader’s sense of humor was evaluated by their employees.

The second limitation is that the sample was obtained from a single IT company in China; this limits the diversity of the sample. Of course, conducting this study in one organization did allow for control of potential organization-level confounding variables. However, this also limits the universality of our model. Future research should sample from a wide range of sources, including from different industries and different regions.

Third, supervisor-subordinate dyadic tenure was not found to act as a moderator, as was hypothesized. This may because leader’s sense of humor is relatively easy to detect at first glance. Thus, enhancement of EPC may occur quickly in the dyadic relationship. In that case, the time employee work with their direct leader may be of little significance. There are other situational innovation atmosphere, etc. Further, creativity is contingent on a variety of individual differences, such as family background, creative self-efficacy, personality, knowledge, and skills. Future research should take these related variables into consideration and apply them to our model.

## Data Availability

All datasets generated for this study are included in the manuscript and/or the supplementary files.

## Ethics Statement

This study was conducted in accordance with the ethical guidelines of the Institutional Review Board of Zhejiang University of Technology (ZJUT) in China, with written informed consent from all subjects. All the employees participated in the survey voluntarily. The protocol was approved by the Institutional Review Board of ZJUT and the Secretariat of Academic Committee of ZJUT, with the permit number 2018001.

## Author Contributions

ZL provided the resource, designed the research, and wrote the first draft of the manuscript. LD collected the data, performed the statistical analysis, and wrote the first draft of the manuscript. TC helped to develop the hypotheses and revised the manuscript. MR revised and copyedited the manuscript.

### Conflict of Interest Statement

The authors declare that the research was conducted in the absence of any commercial or financial relationships that could be construed as a potential conflict of interest.
